# Influence of motivation and a new digitized training program on undergraduate dental students during preclinical scaling training

**DOI:** 10.1186/s12903-020-01343-9

**Published:** 2020-11-30

**Authors:** Miriam Seidel, Simone Sutor, Jonas Conrad, Anne Sophie Engel, Antje Geiken, Sonja Sälzer, Christian Graetz

**Affiliations:** Clinic of Conservative Dentistry and Periodontology, University of Kiel, Kiel, Germany

**Keywords:** Scaling, Nonsurgical periodontal therapy, Subgingival hard deposits, Training evaluation, Biofilm removal

## Abstract

**Background:**

The current study evaluated whether a new digitized scaling training program (DTP: n = 30; supervisor-student-ratio 1:10) improves the performance of undergraduate dental student during a preclinical course in regard to two different instruments [sonic scalers (AIR) and Gracey curettes (GRA)] compared to a conventional training program (CTP: n = 19; supervisor-student-ratio 1:4).

**Methods:**

All the participants received a two-hour lecture on both instruments, followed by a 12-week period with a weekly training program lasting 45 min (10 sessions); one group was supported by DTP. At the end of the training phase, all the participants performed the subgingival scaling of six equivalent test teeth using GRA and AIR. Treatment time, proportion of removed simulated biofilm (relative cleaning efficacy, RCE-b) and hard deposits (RCE-d) were recorded. By using a pseudonymized questionnaire with a 5-point Likert scale, self-assessment of scaling effort, handling, root surface roughness/destruction and effectiveness were evaluated. In addition, personal data such as age, gender, handedness, regularity of playing computer games/consoles and previous dental/technical or medical education were elevated and correlated with cleaning efficacy.

**Results:**

The DTP participants showed higher effectiveness in RCE-b compared to those who used the CTP with GRA (71.54% vs. 67.23%, p = 0.004) and AIR (71.75% vs. 62.63%, p ≤ 0.001), and the DTP students were faster with both instruments (p ≤ 0.001). For RCE-d, there was no significant difference between the DTP and CTP groups (GRA p = 0.471; AIR p = 0.158), whereas DTP showed better RCE-d results with GRA versus AIR (84.68% vs. 77.85%, p < 0.001). According to the questionnaire, no significant differences were detected between the training groups in terms of self-assessment, handling, treatment time, root surface roughness/destruction or effectiveness of the instruments. The CTP group favored AIR compared to GRA regarding the fatigue effect. The CTP and playing computer games/consoles regularly was correlated with lower RCE-b, whereas previous education in medicine/dentistry was correlated with higher RCE-b values.

**Conclusions:**

Within the limitations of the study, the DTP with a reduced supervision effort compared to the CTP resulted in higher effectiveness and lower instrumentation time for removing simulated biofilms.

## Background

The main proceeding of an efficacious cause-related periodontal therapy is the subgingival removal of biofilms and/or hard deposits (mineralized biofilms) [1, 2]. The treatment effect is consistent, irrespective of the choice of instrument (sonic/ultrasonic vs. hand) or mode of delivery (full-mouth vs. quadrant) [3]. Dental students need intensive training in manual skills on manikin heads under clinical conditions before working in real clinical conditions [4–6]. Efforts have been made to improve the efficiency and precision of complex procedures in medicine. With the help of virtual planning visualization, the complex anatomy of the human body is easier to understand [7]. Correspondingly, it seems that conventional techniques have begun to move into the research background. However, until now, these techniques have remained predominant in routine practice [8]. Therefore, it is not surprising that the European Federation of Periodontology (EFP) recommends in their clinical practice guidelines [1] the proper way for a basic dental program to perform conventional scaling and prophylactic procedures. The challenge is to correctly and effectively apply suitable instruments and to positively support the learning process by motivating and strengthening personal drive [9]. Although artificial models do not simulate a perfect realistic situation, training is an important step in reducing treatment time and improving results [10]. In previous studies from our dental school (University of Kiel), the learning curves of scaling with Gracey curettes (GRA) and power-driven instruments using manikin heads with a conventional training program (CTP) [10] or with the help of a self-developed systematic digitized and interactive training program (DTP) [11] have been assessed. We assume that new digital interactions might have an effect on the motivation and self-assessment of the participants. Both studies on the two training concepts are based on the same current state of evidence regarding the choice of hand instruments. However, the investigation of CTP published nearly 20 years ago concerned power-driven instruments (Periopolisher System, Hawe Neos Dental, Bioggio, Schweiz) and not a sonic scaler, as is used in the DTP study. These instruments were common in scaler systems but are no longer marketed today. Thus, we were not able to directly compare both training concepts with regard to effectiveness and motivational effects.

## Methods

The aim of the study was to evaluate the training effect of CTP and DTP with regard to scaling with either a sonic scaler (AIR) or GRA. Furthermore, the participants of both training concepts assessed themselves with regard to their subgingival scaling effort, treatment time, handling, root surface roughness/destruction and effectiveness.

Forty-nine participating students were without randomization assigned, (1) in their 7th semester in the autumn of 2018 to the CTP group, while the students present in the spring of 2019 were assigned to the DTP group. In the DTP group, one student refused to take part in the study (n = 30). In the CTP group, one student failed to finish the training phase (no data at the test visit because of breaks during the preclinical course) (n = 19).

Data for all the participants regarding age, gender, handedness, regularity of playing computer games/consoles and previous dental/technical or medical education were retrieved pseudonymously.

### Experimental setup

During the practical evaluation, with an equivalent structure for all participants, artificial root surfaces were instrumented either with GRA normal shapes 5/6, 7/8, 11/12 and 13/14 (American Eagle Instruments, Missoula, MT, USA) or AIR (Synea, W&H, Bürmoos, Austria) with air pressure and water cooling (30 ml/min), as recommended by the manufacturer at level 2, i.e., "medium" amplitude, combined with a straight, right and left curved, slender tip (1AP, 2APr, 2APl, W&H, Bürmoos, Austria). Simultaneously, a total of 14 questions per participant had to be answered. Four of the fourteen questions were recorded separately for AIR and GRA, resulting in 18 questions in total. In addition, the instrumentation time of each participant was recorded for the two different instruments.

### Student training program and instrumentation procedures

At the beginning of the study, all the participants received a two-hour theoretical introduction to both instruments and to one of the two training programs, either CTP or DTP, but without practical exercises.

The participants in the DTP group received training for both types of instruments, with 10 sessions of 45 min each over a period of 12 weeks. DTP is intended to support the teaching of the necessary work steps and ergonomic aspects for each instrument separately, e.g., with animated GIF (graphics interchange format) or short video sequences to explain the entire construction, technique and seating position of the participants and patient. Therefore, the number of staff and time of supervision (three periodontists with board certificates; one out of ten students) could be reduced compared to the CTP group.

The CTP group also received systematic training over the same period of time according to the continuously modified conventional teaching program from 1994 [10]. This group was supervised and monitored by five periodontists with board certificates (one per four students) without any digital interaction (e.g., no possibility for self-performed working steps or self-control of handling).

In addition, all 49 participants were clinically calibrated during the training courses with regard to application pressure (3–5 N for GRA and < 1 N for AIR). No measurements of root surface destruction or roughness were made.

After 12 weeks (end of the training period), the effectiveness of all the participants (DTP/CTP) was evaluated, and their self-assessment was determined by questionnaire. During practical evaluation, 12 comparable test teeth had to be instrumented with GRA (tooth: 11, 14, 16, 31, 37, 45) and with AIR (tooth: 21, 25, 17, 34, 36, 43). The sequence of both instruments was initially randomized (Microsoft Excel 16, Microsoft Corporation, One Microsoft Way Redmond, WA, USA) for each participant. The participants were asked to instrument the test teeth until they reached the subjective maximum elimination of hard and soft simulated deposits. For each participant, the time required to treat the six teeth per instrument was recorded. The time for changing instruments was considered. Further details including timetables, etc. have been described in detail elsewhere [11].

### Manikin heads, test teeth and planimetric evaluation

All participants performed subgingival scaling on similar manikin heads, which were equipped with modified periodontitis models (Frasaco, Tettnang, Germany). The models exhibited pronounced periodontitis with moderate to advanced horizontal bone loss and isolated and deep vertical pockets. Consequently, the difficulty of instrumenting the teeth differed regarding both anatomy and accessibility. The mean (SD) pocket probing depth (PPD) was 5.8 (2.1) mm (range, 3–11 mm). The test teeth were coated with a thin layer of transparent fluorescent varnish (Shiny White, Rival de Loop Young, Berlin, Germany) between the artificial cementoenamel junction and the alveolar bone. Adhering plaque was simulated with commercial varnish (A-CK, Frasaco, Tettnang, Germany), modified by the ratio of varnish to thickener to simulate the adhesion of subgingival hard deposits. Details of the reproducible and standardized procedure for coating and planimetric evaluation of cleaning efficiency have been described in detail elsewhere [11, 12].

### Questionnaire

During the practical evaluation visit at the end of the 12-week training phase, a questionnaire had to be answered. Before the test, all the participants had to give six statements, rated on a response scale ranging from 1 to 5, according their own personal assessment of the two different scaling instruments used (Additional file 1). Furthermore, four questions aimed at the fatigue effect, treatment time requirement, handling and effectiveness and had to be answered after using each group of scaling instruments (Additional file 2). The evaluation was performed pseudonymously and assigned to a processed model.

### Outcomes

The effectiveness (cleaned area in %) and the treatment time when using GRA or AIR were analyzed in relation to the DTP or CTP group. This study focuses on the evaluation of the questionnaires (personal assessments of the participants). In particular, the training groups (DTP vs. CTP) and GRA versus AIR were compared according to the individual statements on a 5-point Likert scale, differentiated in 25% steps. The answers of each group were counted for every possible answer.

### Statistical analysis

As the aim of the study was to compare two different teaching concepts during a regularly running course, participants were selected consecutively. Hence, a power calculation was not feasible. Data acquisition and collection were performed with Microsoft Excel (Microsoft Excel 16, Microsoft Corporation, One Microsoft Way Redmond, WA, USA). Tables were created and entered into SPSS Statistics (SPSS Statistics 24, IBM, Chicago, IL, USA) for statistical analysis. The normal distribution of questions 1 to 4 and statements 1 to 6 within the two different groups (DTP vs. CTP) between AIR and GRA were tested by the Kolmogorov–Smirnov and Shapiro–Wilk tests. For all questions and statements, there was no normal distribution. Subsequently, a mean value comparison was performed using the Mann–Whitney U-test, and significant differences were found both within the DTP and CTP groups with regard to the AIR versus GRA group. A linear regression model was constructed using RCE-b after treatment as the dependent variable, while age, gender, handedness (right/left/two-handed), playing computer games/consoles regularly (no/yes), previous education in medicine/dentistry (no/yes), instrument (AIR/GRA) and group of training (DTP/CTP) were the independent variables. All tests were two-sided; statistical significance was assumed if p ≤ 0.05.

## Results

### Participants’ characteristics

All students participating in the study were in their 7th semester at the Kiel Dental School; there were 30 students in the DTP group and 19 students in the CTP group. The majority of the participants were right-handed, and approximately twice as many students in the DTP group had previous medical/dental education (Table 1).

**Table 1:** Participants’ characteristics for the DTP group (DTP: digitized training program) and CPT group (CTP: conventional training program)

	DTP concept (Graetz et al. [11])	CTP concept
Sex (female/male)	21/9	15/4
Mean (SD) age in years	25.50 (4.11) [21–42]	27.16 (5.71) [22–42]
N. of participant with a previous education in medicine/dentistry	11	3
Handedness (N of participants who were right/left/two-handed)	24/5/1	18/1/0
N of participants who played computer games/consoles regularly	6	3

### Removal of simulated biofilm and hard deposits—effectiveness and treatment time

The DTP group had significantly more effective RCE-b results than the CTP group with AIR (p < 0.001) as well as with GRA (p = 0.004). At the tooth level, using GRA in molars led to significantly better RCE-b results in both groups (DTP vs. CTP) compared to AIR. Using GRA, the CTP participants showed higher RCE-b results than the DTP participants [79.82 (20.85) vs. 72.08 (23.35); p = 0.001]. Conversely, for AIR, the DTP group cleaned molars with higher effectiveness [64.95 (24.10) vs. CTP 56.78 (28.73); p = 0.003].

Using GRA, the CTP group removed artificial biofilms best in molars compared to anterior teeth and premolars (p ≤ 0.001), but there were no such differences for the DTP group in RCE-b (p > 0.05; Table 2). Using AIR, the DTP group showed better cleaning results for premolars than for anterior teeth and for last molars (p ≤ 0.05), whereas the CTP participants removed more artificial biofilms in anterior teeth and premolars [67.28 (26.11) and 64.06 (29.14); p = 0.318] than molars [56.78 (28.73), p ≤ 0.05].

**Table 2:** Efficacy of removed simulated biofilm (RCE-b in %) and hard deposits (RCE-d in %) with Gracey curettes (GRA) or sonic scaler (AIR) and the treatment time (in min) for all 12 test teeth after training phase either with digitized training program (DTP) or conventional training program (CTP)

Variable	DTP concept (Graetz et al. [11])	CTP concept	Differences between groups of training concepts
Overall RCE-b in %^a^			
GRA	71.54(23.90)	67.23 (24.72)	p = 0.004
AIR	71.75(23.05)	62.63 (28.34)	p < 0.001
Differences between groups of instruments	p = 0.864	p = 0.011	
RCE-b in % anterior teeth			
GRA	71.14 (26.36)	65.51 (22.88)	p = 0.039
AIR	69.19 (22.59)	67.28 (26.11)	p = 0.454
Differences between groups of instruments	p = 0.401	p = 0.541	
RCE-b in % premolar			
GRA	71.35 (22.09)	56.07 (24.53)	p < 0.001
AIR	81.28 (19.0)	64.06 (29.14)	p < 0.001
Differences between groups of instruments	p < 0.001	p = 0.013	
RCE-b in % molar			
GRA	72.08 (23.35)	79.82 (20.85)	p = 0.001
AIR	64.95 (24.10)	56.78 (28.73)	p = 0.003
Differences between groups of instruments	p = 0.001	p < 0.001	
Overall RCE-d in %^a^			
GRA	84.68 (16.84)	88.35 (11.46)	p = 0.471
AIR	77.85 (13.98)	83.46 (11.69)	p = 0.158
Differences between groups of instruments	p < 0.001	p = 0.201	
Treatment time in min per 6 test teeth per group of instruments			
GRA	16.67 (3.31)	22.58 (4.20)	p < 0.001
AIR	19.80 (4.52)	22.26 (3.81)	p < 0.001
Differences between groups of instruments	p < 0.001	p = 0.236	

Average values (SD) and p-values. Data for RCE-d on tooth level were not available

^a^(N of RCE-b in total 720 and N of RCE-d in total 420)

Contrary to the results for RCE-b, no significant difference for RCE-d between both groups of training concepts was measured. The DTP group showed significantly more effective RCE-d results with GRA versus AIR [84.68 (16.84) vs. 77.85 (13.98); p < 0.001]. In the CTP group, no significant difference between the instruments was detectable (Table 2).

In total, in the DTP group, 66.7% (n = 20) reached the goal of cleaning ≥ 70% using GRA for RCE-b versus 36.9% (n = 7) in the CTP group. A similar result for RCE-b was found using AIR, with 63.3% (n = 19) for the DTP group versus 21.1% (n = 4) for the CTP group. For RCE-d with GRA, 83.4% (n = 25) of the DPT participants were able to clean simulated hard deposits with an efficacy of ≥ 70% (AIR: 80.0%, n = 24). No difference between both instruments was found in the CTP group; as with both instruments, 89.5% (n = 17) of all participants reached this goal.

Treatment time was significantly shorter in the DTP group with both types of instruments (p < 0.001, Table 2).

The results of a linear regression model (Table 3) showed a direct correlation between previous education in medicine/dentistry and RCE-b (p = 0.050) and an inverse correlation between playing computer games/consoles regularly and RCE-b (p = 0.025). Furthermore, the training concept in CTP correlated with lower RCE-b values (p = 0.017).

**Table 3:** Results of linear regression analysis for the significant predictors of efficacy to remove simulated biofilms (RCE-b)

Predictors for RCE-b in all participants	B	SE	95% CI		p-value
			Lower limit	Upper limit	
Constant term	75.594	7.181	61.328	89.860	0.000
Age	− 0.229	0.224	− 0.673	0.216	0.309
Gender	4.695	2.400	− 0.072	9.462	0.053
Handedness	3.517	2.198	− 0.849	7.883	0.113
Plays computer games/consoles regularly	− 6.371	2.798	− 11.929	− 0.813	0.025
Previous education in medicine/dentistry	4.671	2.353	− 0.004	9.346	0.050
Group of instruments (GRA, AIR)	− 1.594	1.738	− 5.046	1.859	0.362
Group of training (CTP, DTP)	− 4.839	1.997	− 8.807	− 0.870	0.017

*B* regression coefficient, *SE* standard error

### Questionnaire part one: self-assessment before using instruments

The evaluation before instrumentation with GRA or AIR showed that there were no significant differences in the self-assessment according to the six statements (S1, S3–S6) between DTP versus CTP (Table 4). Only for the estimation of tiring during scaling did participants in the CTP group favor AIR (S2, p = 0.003).

**Table 4:** Average values (SD) of statements according handling (S1, S2), treatment time (S3), root surface roughness/root destruction (S4, S6) and effectiveness (S5) by the participants of the digitized training program (DTP) versus conventional training program (CTP) for Gracey curettes (GRA) and for sonic scaler (AIR)

Statements	DTP concept (Graetz et al. [11])	CTP concept	Differences between groups of training concepts
S1 Today, I think that scaling with sonic scaler is more difficult/easier to learn than the scaling with curettes (1 = very difficult to 5 = very easy)	3.53 (0.73)	3.47 (0.75)	p = 0.91
S2 Today, I think that scaling with sonic scaler is less tiring than the scaling with curettes (1 = a lot more tiring to 5 = a lot less tiring)	3.45 (0.81)	4.16 (0.59)	p = 0.003
S3 Today, I think that scaling with sonic scaler is more time-saving/less time-saving than scaling with curettes: 1 = extremely time-saving to 5 = very time-consuming	3.21 (0.96)	3.00 (1.08)	p = 0.38
S4 Today, I think that scaling with sonic scaler is more or less gentle on the substance than scaling with curettes (1 = very gentle to 5 = very substance demanding)	2.66 (0.88)	3.11 (0.97)	p = 0.10
S5 Today, I think that scaling with sonic scaler is more effective/less effective than scaling with curettes regarding the whereabouts of hard deposits and biofilm (1 = highly effective to 5 = very ineffective)	3.24 (0.86)	3.05 (0.83)	p = 0.51
S6 Today, I think that scaling with sonic scaler produces rougher/less rough tooth surfaces than scaling with curettes (1 = very rough surface to 5 = very smooth surface)	3.21 (0.96)	2.84 (0.93)	p = 0.24

Average values (SD) and p-values

Overall, all the participants in both training groups estimated more or less balanced results for learning effort, treatment time, effectiveness, destruction and root surface roughness for GRA versus AIR (S1, S3–S6).

### Questionnaire part two: self-assessment after using instruments

The evaluation after using both types of instruments showed that there were no significant differences in the self-assessment regarding DTP versus CTP (Table 5). However, in the intragroup analysis, the CTP participants favored AIR regarding fatigue (Q1), handling (Q3) and the self-estimated effectiveness in molars (Q4 molar). In contrast, the participants who had received training with DTP favored GRA according to treatment time compared to AIR (Q2, Table 5).

**Table 5:** Average values (SD) of answers according to fatigue effect (Q1), treatment time (Q2), handling (Q3) and effectiveness (Q4 in %, separated for anterior teeth, premolars and molars) by the participants of the digitized training program (DTP) versus conventional training program (CTP) for Gracey curettes (GRA) and for sonic scaler (AIR)

Questions	DTP concept (Graetz et al. [11])	CTP concept	Differences between groups of training concepts
Q1 How did you feel scaling with the instrument? (1 = very tiresome/strenuous to 5 = extremely easy)			
GRA	3.00 (1.05)	2.56 (0.78)	p = 0.129
AIR	3.27 (1.08)	3.72 (1.02)	p = 0.132
Differences between groups of instruments	p = 0.32	p = 0.007	
Q2 What was your sense of time when scaling with the instrument? (1 = highly time-consuming to 5 = highly time-saving)			
GRA	3.34 (0.89)	3.00 (0.69)	p = 0.067
AIR	2.70 (0.99)	3.06 (1.06)	p = 0.286
Differences between groups of instruments	p = 0.016	p = 0.768	
Q3 How did you find the handling of the instrument? (1 = extreme complex to 5 = quite simple)			
GRA	3.40 (0.76)	3.11 (0.76)	p = 0.215
AIR	3.37 (0.85)	3.67 (0.77)	p = 0.240
Differences between groups of instruments	p = 0.796	p = 0.038	
Q4a self-estimated effectiveness for anterior teeth [0% = 1 (no biofilm and hard deposits removed) to 100% = 5 (completely removed)]			
GRA	4.0 (0.64)	4.17(0.71)	p = 0.391
AIR	3.80 (0.55)	4.0(0.34)	p = 0.151
Differences between groups of instruments	p = 0.109	p = 0.317	
Q4b self-estimated effectiveness for premolar [0% = 1 (no biofilm and hard deposits removed) to 100% = 5 (completely removed)]			
GRA	3.93 (0.64)	4.06 (0.64)	p = 0.519
AIR	3.77 (0.73)	3.83 (0.62)	p = 0.784
Differences between groups of instruments	p = 0.059	p = 0.096	
Q4c self-estimated effectiveness for molar [0% = 1 (no biofilm and hard deposits removed) to 100% = 5 (completely removed)]			
GRA	3.60 (0.73)	3.94 (0.64)	p = 0.097
AIR	3.50 (0.73)	3.61(0.50)	p = 0.537
Differences between groups of instruments	p = 0.405	p = 0.008	

Average values (SD) and p-values

## Discussion

In the present study, we found that the DTP participants were 5–10% more efficient and needed less time for RCE-b with both groups of instruments than were CTP participants. Additionally, we identified two predictors of successful cleaning: DTP (p = 0.017) and pre-education in medicine/dentistry (p = 0.05). In contrast, regular computer/console play (p = 0.025) worsened the cleaning performance of our undergraduate students.

However, both training groups were similarly efficient for RCE-d, i.e., 78–88% (Table 2). A possible reason for the higher effectiveness for RCE-b but not for RCE-d would be the tactile dedication of hard deposits. Hence, systematic work is of less importance than for the removal of biofilms. In particular, curettes allow tactile control [13]. In addition to standardized working parameters [14], the efficacy of biofilm removal depends on experience [10, 12]. Another unusual significant difference for RCE-b was found at the tooth level, as the CTP group was more effective in the molar region with GRA than the DTP group (RCE-b: 79.82 (20.85)% versus 72.08 (23.35)%, p = 0.001). Furthermore, against current knowledge [15], GRA leads to better cleaning efficacy in molars in both training groups. An explanation could be that only the outer root surfaces but not the furcation area of the involved molars were assessed in the current study. Hence, accessibility for both types of instruments was equally good [16].

The total training phase took the same amount of time, but the training with DTP needed less supervision time by fewer supervisors (DTP in the ratio 1:10 versus CTP in the ratio 1:4), which was one goal of developing DTP.

In general, the effectiveness of subgingival scaling depends not only on the type of instrument and the anatomical conditions (e.g., tooth type) but also on the students’ skills in handling the instruments. Other studies have considered differences in the participant's experience and relate them to the success of treatment [17, 18]. High motivation seems to be a further prerequisite for improved performance. For example, the study by König, Ruhling [19] showed that motivated groups achieve on average approximately 25% higher cleaning performance when removing simulated deposits than groups that were less motivated during training. The authors showed that students who had received very comprehensive support were much more realistic about their own effectiveness. In contrast, the less motivated group overestimated their own results by more than 20% [19]. In the current study, a similar effect of motivation by DTP can be assumed, whereby the benefit is essentially lower in total and more specifically distributed for the two different groups of instruments (Table 2).

The decision of which instrument for subgingival scaling should be used has to be placed in the hands of the operator. The choice should be based upon the experience, skills and preference. However, to make the decision, a realistic self-assessment is of major importance. Expectations of success and willingness to perform well are relevant factors among the many aspects of performance motivation [20]. An operator who does not expect to be successful is not motivated to perform well. From the point of view of learning psychology, performance motivation depends on intrinsic and extrinsic factors [1]. Intrinsic motivation seems to be particularly important for learning. It leads to more intensive information processing, which depends on systematic knowledge, conceptual understanding and the recognition of superordinate relationships. In addition to the mentioned goal of reducing the necessary effort for teaching, another goal of DTP was to strengthen the motivation of undergraduate students. Self-assessment of one's own performance and feedback from the instructors appear to be essential components of a formative learning process and the basis for further development. As described above, all the participants were asked to use a questionnaire to assess how they felt when working with GRA and AIR or how efficiently they thought they were working. We showed that the participants of the more self-reliant DTP group more often assessed their effectiveness and time requirements more realistically than did the other participants. However, the participants in the CTP group preferred AIR for higher effectiveness.

Previously, we found that the learning curve for RCE-b was steeper with AIR than with GRA in DTP [11]. Without training, only 27% of all the students reached a cleaning performance of at least 70% when using AIRs (GRA: 40%). We have already observed a similar phenomenon in previous studies [10, 12]. A possible explanation might be the prior knowledge of using a universal curette and inexperience in using powered equipment. Furthermore, this could also justify why the CTP group preferred the AIR rather than the DTP group, which also judged the mechanical instrument to be more effective and less tiring (Table 4). At the end of the training phase by DTP, 30% removed at least 70% of simulated biofilm with GRA compared to the participants in the CTP group. When using AIR, this difference was approximately 42%. Interestingly, for RCE-d, an almost similar percentage of participants of 80–90% removed hard deposits with an efficacy of ≥ 70%. Therefore, it should be mentioned that (1) hard subgingival deposits are always easier to detect as biofilms and (2) it is important to learn tactility for scaling.

In addition, the CTP group stated before the evaluation that they considered using AIR less tiring than the DTP group. After using both types of instruments, the DTP group assessed themselves in the same way as the CTP group (statistically no differences for GRA/AIR). However, the CTP participants overestimated their own effectiveness, as they worked significantly less efficiently with both instruments (p ≤ 0.01) than the DTP group. Additionally, the CTP group was slower but estimated the required time analogously to the DTP group (GRA p = 0.067 and AIR p = 0.028). This estimation can possibly be explained by the fact that the CTP group is not as proficient in the use of GRA as the DTP group. Hence, it can be concluded that using AIR is less tiring and has a lower learning curve. Participants in the DTP group recognized that the use of AIRs takes more time when used correctly and can be perceived as tiring (and noisy) over this period of time. In addition, the self-assessment in DTP might be better, as the participants could rely less on support from supervisors and hence had to help themselves or help each other more often.

The most important limitation of our simulation is that the results cannot be transferred to a clinical setting directly [17]. RCE can only be assessed in an in vitro setup with simulated biofilms and hard deposits on artificial teeth. Corresponding to our earlier study regarding the learning curve by DTP [11], DTP seems to be helpful for inexperienced users. Our study evaluated DTP versus CTP in routine teaching. We did not focus on the best possible effectiveness of scaling by undergraduate students or measuring side effects such as root destruction [21].

However, the effect of digital media on training in dentistry should not be overestimated. Both an innovative teaching concept and the ability to realistically self-assess performance play important roles in the learning process, similar to other factors (general stress in studies, different teaching methods, etc.) [19]. Overall, the additional effort for digitizing teaching (e.g., higher costly technical equipment, time to organize) pays off in terms of both effectiveness and time expenditure by higher motivation of the students and lower supervision key.

## Conclusions

Within the limits of the study, it can be concluded that DTP is helpful in training subgingival scaling in undergraduate dental students. The DTP participants assessed themselves more realistically, and they cleaned significantly better overall than did the CTP group. A further improvement and even a permanent increase in motivation might be achieved by a direct digital feedback system or with, e.g., personalized digital animation, which must be evaluated in future studies.

## Supplementary information

**Additional file 1**. Six statements were classified on a 5-point Likert scale by each participant before practical training.

**Additional file 2**. After using Gracey curettes and sonic scalers, each participant had to answer four questions for each group of instruments separately.

## Data Availability

The datasets used and analyzed during the current study are not publicly available due [national data protection law] but are available from the corresponding author on reasonable request.

## Abbreviations

AIR: Sonic scaler
CTP: Conventional scaling training program
DTP: Digitized scaling training program
GRA: Gracey curettes
RCE-b: Relative cleaning efficacy of biofilm
RCE-d: Relative cleaning efficacy of hard deposits
