# Supplementary material for: Influence of motivation and a new digitized training program on undergraduate dental students during preclinical scaling training
Source: BMC Oral Health. 2020 Nov 30;20:346. doi: 10.1186/s12903-020-01343-9 (PMC7706025; doi:10.1186/s12903-020-01343-9)
Supplement: Supplementary file 1 — Additional file 1. Six statements were classified on a 5-point Likert scale by each participant before practical training. [file 12903_2020_1343_MOESM1_ESM.docx]

**Additional files**

**Additional file 1:** Six statements were classified on a 5-point Likert scale by each participant before practical training.

| S1. Today, I think that scaling with sonic scaler is more difficult/easier to learn than the scaling with curettes: 1= very difficult to 5= very easy |
| --- |
| S2. Today, I think that scaling with sonic scaler is less tiring than the scaling with curettes: 1= a lot more tiring to 5= a lot less tiring |
| S3. Today, I think that scaling with sonic scaler is more time-saving/less time-saving than scaling with curettes: 1= extremely time-saving to 5= very time-consuming |
| S4. Today, I think that scaling with sonic scaler is more or less gentle on the substance than the scaling with curettes: 1= very gentle to 5= very substance demanding |
| S5. Today, I think that scaling with sonic scaler is more effective/less effective than the scaling with curettes regarding the whereabouts of hard deposits and biofilm: 1= highly effective to 5= very ineffective |
| S6. Today, I think that scaling with sonic scaler produces rougher/less rough tooth surfaces than scaling with curettes: 1= very rough surface to 5= very smooth surface. |
