# Supplementary material for: Influence of motivation and a new digitized training program on undergraduate dental students during preclinical scaling training
Source: BMC Oral Health. 2020 Nov 30;20:346. doi: 10.1186/s12903-020-01343-9 (PMC7706025; doi:10.1186/s12903-020-01343-9)
Supplement: Supplementary file 2 — Additional file 2. After using Gracey curettes and sonic scalers, each participant had to answer four questions for each group of instruments separately. [file 12903_2020_1343_MOESM2_ESM.docx]

**Additional files**

**Additional file 2:** After using Gracey curettes and sonic scalers, each participant had to answer four questions for each group of instruments separately.

| Q1. How did you feel scaling with the instrument? (1= very tiresome/strenuous to 5= extremely easy) |
| --- |
| Q2. What was your sense of time when scaling with the instrument? (1= highly time-consuming to 5= highly time-saving) |
| Q3. How did you find the handling of the instrument? (1= extreme complex to 5= quite simple) |
| Q4. Estimate the effectiveness of your root surface treatment! This question was separated for anterior teeth (Q4a), premolars (Q4b) and molars (Q4c) estimation in 25%-steps: 0%= 1 (no biofilm and hard deposits removed) to 100%= 5 (biofilm and hard deposits completely removed). |
